# Spatial Metabolomics-Based Integrated Analysis Provides Evidence for the Mechanism of Salvianolic Acid A-Induced Mitochondrial Apoptosis in Non-Small Cell Lung Cancer via the ROS-Dependent Caspase Pathway

**DOI:** 10.3390/biom16101434

**Published:** 2026-10-02

**Authors:** Ruyu Jiang, Hairong Xiang, Lushuang Xie, Linfeng Zhao, Dawei Zeng, Yue Zhang, Jiazhen Xie, Zhenxu Pan, Yanju Gong, Lan Yang

**Affiliations:** 1 College of Basic Medicine, Chengdu University of Traditional Chinese Medicine, Chengdu 611137, China; jiangruyu@stu.cdutcm.edu.cn (R.J.);; 2 School of Information and Software Engineering, University of Electronic Science and Technology of China, Chengdu 611731, China

**Keywords:** Salvianolic acid A, spatial metabolomics, mass spectrometry imaging, network pharmacology, non-small cell lung cancer, mitochondrial apoptosis

## Abstract

Salvianolic acid A (SAA) exhibits antitumor activity against non-small cell lung cancer (NSCLC), but the metabolic regulatory mechanisms underlying its effects remain incompletely understood. In this study, we integrated desorption electrospray ionization mass spectrometry imaging (DESI-MSI), network pharmacology, molecular docking, and functional validation to investigate the effects of SAA on NSCLC. SAA inhibited H1299 cell viability, migration, and invasion and induced apoptosis. DESI-MSI analysis of xenograft tumor tissues revealed marked metabolic alterations after SAA treatment, including changes in ceramides, glycerophospholipids, and arachidonic acid candidates. Network pharmacology and molecular docking further suggested the involvement of oxidative stress- and apoptosis-related pathways. Functionally, SAA increased intracellular ROS levels, impaired mitochondrial function, and reduced mitochondrial membrane potential. Treatment with the ROS scavenger NAC partially reversed SAA-induced ROS accumulation, loss of cell viability, and increased levels of cleaved Caspase-9 and Caspase-3. Consistently, Western blotting and immunofluorescence analyses showed concentration-dependent activation of Caspase-9 and Caspase-3, whereas the pan-caspase inhibitor Z-VAD-FMK significantly attenuated SAA-induced cell death and apoptosis. Collectively, these findings provide evidence that SAA induces metabolic remodeling and promotes ROS-associated mitochondrial dysfunction and caspase-mediated apoptosis in NSCLC, thereby suggesting a potential metabolic mechanism underlying its antitumor activity.

## 1. Introduction

Lung cancer has long been a leading cause of cancer-related mortality worldwide, with non-small cell lung cancer (NSCLC) accounting for the majority of lung cancer cases [1]. Although significant progress has been made in targeted therapy and immunotherapy, treatment outcomes are often limited, especially in advanced cases, and a significant proportion of patients experience recurrence or metastasis [2]. Therefore, it is necessary to conduct further research to develop more effective drugs for NSCLC.

Salvianolic acid A (SAA), the most abundant water-soluble compound extracted from *Salvia miltiorrhiza* Bunge, has exhibited antioxidant, anti-inflammatory, and anticancer activity, and recent studies have also highlighted its potential in cancer therapy [3,4,5,6,7]. Previous studies have shown that SAA inhibits angiogenesis by reducing GRP78 release and also suppresses metastasis [8,9]. Furthermore, SAA enhances sensitivity to chemotherapy and counteracts drug resistance [10]. In our recent study, we further demonstrated that SAA induces ferroptosis in NSCLC through the SRC/YAP/GPX4 axis, accompanied by oxidative stress-related changes [11]. However, whether SAA-induced redox and metabolic alterations are also involved in other regulated cell-death processes, particularly mitochondrial apoptosis, remains unclear.

Metabolomics is a technique for studying the composition and changes in endogenous small-molecule metabolites in living organisms. Over the past few years, this method has been used effectively to study the mechanisms of numerous diseases, thereby providing new insights [12]. With technological advancements, spatial metabolomics that preserves in situ information has emerged. This technique combines molecular detection with spatial visualization. Among several MSI techniques, desorption electrospray ionization mass spectrometry imaging (DESI-MSI) operates at atmospheric pressure, requires no matrix, enables direct detection of fully ionized metabolites, and offers extremely high sensitivity [13,14]. Consequently, DESI-MSI has been widely used to identify disease biomarkers and visualize metabolic changes induced by drugs.

In this study, we integrated MSI-based spatial metabolomics, network pharmacology, and molecular docking to investigate SAA-induced metabolic alterations in tumor tissues and their potential relationship with ROS-associated mitochondrial apoptosis. In contrast to our previous work focusing on ferroptosis, the present study investigates apoptosis-related responses from a distinct mechanistic perspective using spatial metabolomics and functional data. Collectively, our findings provide additional insight into the antitumor mechanisms of SAA in NSCLC.

## 2. Materials and Methods

### 2.1. Cell Line and Chemicals

Human NSCLC line H1299 (#CRL-5803) was obtained from the American Type Culture Collection (ATCC, Manassas, VA, USA). Salvianolic acid A (96574-01-5, purity ≥ 98%) was procured from Chengdu Lingliu Biotechnology (Chengdu, China). SAA was dissolved in DMSO to prepare a stock solution. Z-VAD-FMK was purchased from Beyotime Biotechnology, Shanghai, China, C1201, and NAC was purchased from MCE, Monmouth Junction, NJ, USA, 616-91-1. In all experiments, the final DMSO concentration in both the control and treatment groups was maintained at ≤0.1%. The primary antibodies included Ki67 (CST, Danvers, MA, USA, #9449, 1:1000), cleaved Caspase-3 (zenbio, Chengdu, China, 341034, 1:1000) and cleaved Caspase-9 (CST, Danvers, MA, USA, Asp315, 1:1000).

### 2.2. Cell Viability and Proliferation Assay

The CCK-8 assay, wound healing assay, and Transwell assay were performed as described previously [11].

### 2.3. Flow Cytometry

Apoptosis assay and ROS analysis were performed according to previously described methods [11].

### 2.4. Xenograft Model

BALB/c nude mice aged 6–8 weeks (20 ± 2 g), including six males and six females, were sourced from Chengdu yaokang Biotechnology Co., Ltd. (Chengdu, China) (license number: SCXK(Chuan)2025-0034). All animals were housed under SPF-grade laboratory conditions with a 12 h light/dark cycle at 20–26 °C and access to pathogen-free rodent feed and water. Each mouse received a subcutaneous injection of H1299 cells (5 × 10^6^) on the right side. Once the tumor volume reached approximately 50–100 mm^3^, the mice were randomly assigned to two groups using a computer-generated random sequence, with six mice per group. To maintain a balanced sex distribution, each group consisted of three male and three female mice. No animals were excluded after randomization. The experimental group received intraperitoneal injections of SAA (60 mg/kg), while the control group received PBS. Treatment was administered every other day for 16 consecutive days. Tumor length and width were measured every 2 days, and tumor volume was calculated using the formula: Volume = (Length × Width^2^)/2. At the end of the treatment period, the mice were euthanized and the tumors were collected for subsequent analysis. All outcome assessments and data analyses were performed by investigators blinded to group allocation. The sample size of six mice per group was selected as the minimum number required to detect biologically meaningful differences while minimizing animal use in accordance with animal-welfare considerations and ethical-review requirements. All animal studies were performed according to a protocol approved by the Animal Ethics Committee of Chengdu University of Traditional Chinese Medicine on 15 December 2025 (Approval No.: 2025806).

### 2.5. Sample Preparation

Tumor tissues from six independent mice in each group were used for MSI analysis. Each tumor was cryosectioned at −20 °C using a cryostat (Taiva, Xiaogan, China, TL-820E) to obtain serial sections with a thickness of 10 μm. One intact section with a relatively large tumor cross-sectional area and without obvious folding or tearing was selected from each tumor for MSI analysis. The selected sections were vacuum-dried at −20 °C for 1 h, followed by drying at room temperature for 2 h prior to MSI analysis. All sections were prepared and processed under identical conditions. The order of sample preparation and MSI acquisition was not formally randomized.

### 2.6. DESI-MSI Analysis

Spatial metabolomics profiling was performed using a SYNAPT G2 QTOF mass spectrometer equipped with a DESI ion source and a nano UPLC system (Waters Corporation, Milford, MA, USA). Full-scan MS data were acquired over an *m*/*z* range of 50–1200 in both positive and negative modes. The solvent was ACN:H_2_O (95:5, *v*/*v*) with a flow rate at 1.5 μL/min both in positive and negative modes. The incidence angle of the DESI sprayer relative to the sample surface was 75°. The sprayer-to-inlet capillary distance, sprayer-to-sample distance, and sample-to-inlet capillary distance were maintained at 5.0, 1.5, and 1.5 mm, respectively. The source temperature and extended inlet-capillary temperature were maintained at 120 °C and 300 °C, respectively, and the capillary voltage was set to 0.6 kV. The spatial resolution was set to 100 μm × 100 μm and stepped in 400 μm increments along the y-axis. All tissue sections were analyzed using identical instrumental settings. The order of sample preparation and MSI acquisition was not formally randomized. Raw mass spectrometry data were processed using HDI 1.8 software (Waters, USA). For all tissue sections, a region of interest (ROI) was designated for each intact section, and the data were normalized using total ion current (TIC).

### 2.7. Data Analysis

Metabolite annotation was performed using MSI Analyte Browser 2.1.0 with the Human Metabolome Database (HMDB). The mass tolerance was set as 5 ppm. Accurate-mass database matching alone was considered a putative annotation and was not regarded as unequivocal structural identification. The processed data were analyzed using MetaboAnalyst 6.0 (https://www.metaboanalyst.ca/, accessed on 13 July 2026). PCA and OPLS-DA were performed to visualize global metabolic differences between the control and SAA-treated groups. Differential metabolic features were screened using *VIP* ≥ 1, *p* < 0.05, and fold change (*FC*) ≥ 1.2 or *FC* ≤ 0.8. Volcano plots were used to visualize the magnitude and statistical significance of metabolic alterations. The confidence of metabolite annotation was assessed according to the five-level framework originally proposed for high-resolution mass spectrometry and subsequently adapted for mass spectrometry imaging [15]. In this framework, Level 4 represents an unequivocal molecular formula supported by the available high-resolution MS information, without unique structural confirmation, whereas Level 5 represents an accurate-*m*/*z* feature for which the available evidence does not support an unequivocal molecular formula. No authentic reference standards, diagnostic MS/MS spectra, or MRM transitions were used to confirm the selected differential MSI features in the present study. Therefore, none of the reported annotations were assigned to Levels 1–3. Features for which the available high-resolution MS data supported an unequivocal molecular formula were classified as Level 4, whereas features with multiple plausible molecular formula/adduct assignments were classified as Level 5. The confidence levels for the selected features are provided in Supplementary Table S3. For lipid-related features, structural annotations were restricted to the highest level supported by the available mass-spectrometric evidence [16]. Differential metabolic features were subsequently used for KEGG pathway enrichment analysis.

### 2.8. Network Pharmacology Analysis and Molecular Docking

Network pharmacology analysis was performed as previously described [11]. For molecular docking, the 3D structures of the selected target proteins were retrieved from the RCSB Protein Data Bank, and the molecular structure of SAA was obtained from the PubChem database. The PDB IDs of the target proteins are provided in Supplementary Table S4. Next, the co-crystallized ligands, crystallographic water molecules, and other nonessential heteroatoms were removed from the protein structures using PyMOL software, and the receptor and ligand structures were prepared using AutoDockTools 1.5.7, including the addition of hydrogen atoms, assignment of partial atomic charges, definition of ligand rotatable bonds, and conversion to PDBQT format. AutoDock Vina 1.2.2 was employed for molecular docking with the receptor treated as rigid and the ligand treated as flexible. The docking search space was individually defined for each target protein, and the corresponding box-center coordinates, box dimensions, docking scores, number of runs, and docking parameters are summarized in Supplementary Table S4. PyMOL 2.2.0 was used to visualize the optimal protein–ligand complexes generated from these processes.

### 2.9. ROS Staining

H1299 cells were incubated in serum-free medium containing DCFH-DA (Solarbio, Beijing, China, CA1410) at 37 °C in the dark for 30 min. Finally, the cells were imaged under a confocal fluorescence microscope (ImageXpress Micro Confocal, San Jose, CA, USA).

### 2.10. MitoSOX Staining

H1299 cells were incubated with 5 μM MitoSOX Red (Beyotime Biotechnology, Shanghai, China, S0061S) at 37 °C for 30 min in the dark, followed by nuclear staining with Hoechst 33342. Finally, the cells were imaged under a confocal fluorescence microscope (ImageXpress Micro Confocal, San Jose, CA, USA).

### 2.11. MitoTracker Staining

Live cells were incubated with 100 nM MitoTracker Red (Beyotime Biotechnology, Shanghai, China, C1049B) working solution at 37 °C for 30 min. Fluorescence images were acquired using an Olympus BX53 fluorescence microscope (Olympus, Tokyo, Japan) with a 40× objective. All images were acquired under identical exposure and gain settings.

### 2.12. JC-1 Measurement

The JC-1 mitochondrial membrane potential kit (Solarbio, Beijing, China, M8650) was used to assess mitochondrial membrane potential. Briefly, after 24 h of H1299 treatment, the cells were digested with trypsin and incubated with 5 µg/mL JC-1 in the dark for 30 min. The cell suspension was then washed twice with serum-free medium and resuspended in PBS, followed by quantitative analysis using a BD FACSCanto II flow cytometer (BD FACSCanto II, Franklin Lakes, NJ, USA).

### 2.13. Western Blot

Western blot was performed using standard procedures, including protein extraction with RIPA lysis buffer, protein quantification by the BCA assay (Beyotime Biotechnology, Shanghai, China, P0010), SDS-PAGE, PVDF transfer (Merck, Darmstadt, Germany, ISEQ00010), and antibody incubation. Finally, the results were visualized using a chemiluminescence imaging system.

### 2.14. Immunofluorescence Staining

Immunofluorescence staining was carried out following the reference’s protocol instructions [11].

### 2.15. Hematoxylin and Eosin Staining

At the end of the experiment, tumor tissue was harvested from the mice, fixed in 4% paraformaldehyde, embedded in paraffin, and sectioned. The sections were stained with hematoxylin, followed by eosin staining. After clearing, the sections were mounted with neutral resin. Histopathological changes were evaluated under a light microscope (Leica, Wetzlar, Germany, DM500).

### 2.16. Immunohistochemistry Staining

Immunohistochemical staining was performed according to previously described procedures [14].

### 2.17. Statistical Analysis

All statistical analyses were performed using GraphPad Prism 10.0. Independent biological replicates were considered the statistical units, whereas technical replicates were treated as repeated measurements and were not considered independent samples. In vitro experiments were performed using at least three independent biological replicates unless otherwise stated. For animal experiments, each mouse was considered an independent biological replicate, and the xenograft experiments included six mice per group. Data are presented as mean ± standard deviation (SD). Comparisons between two groups were performed using a two-tailed Student’s *t*-test. Comparisons among multiple groups were performed using one-way analysis of variance (ANOVA) followed by Tukey’s multiple-comparison post hoc test. For repeated measurements, repeated-measures ANOVA was applied where appropriate. Statistical significance was defined as *p* < 0.05.

## 3. Results

### 3.1. SAA Inhibits Proliferation and Induces Apoptosis in H1299 Cells

**Figure 1:**
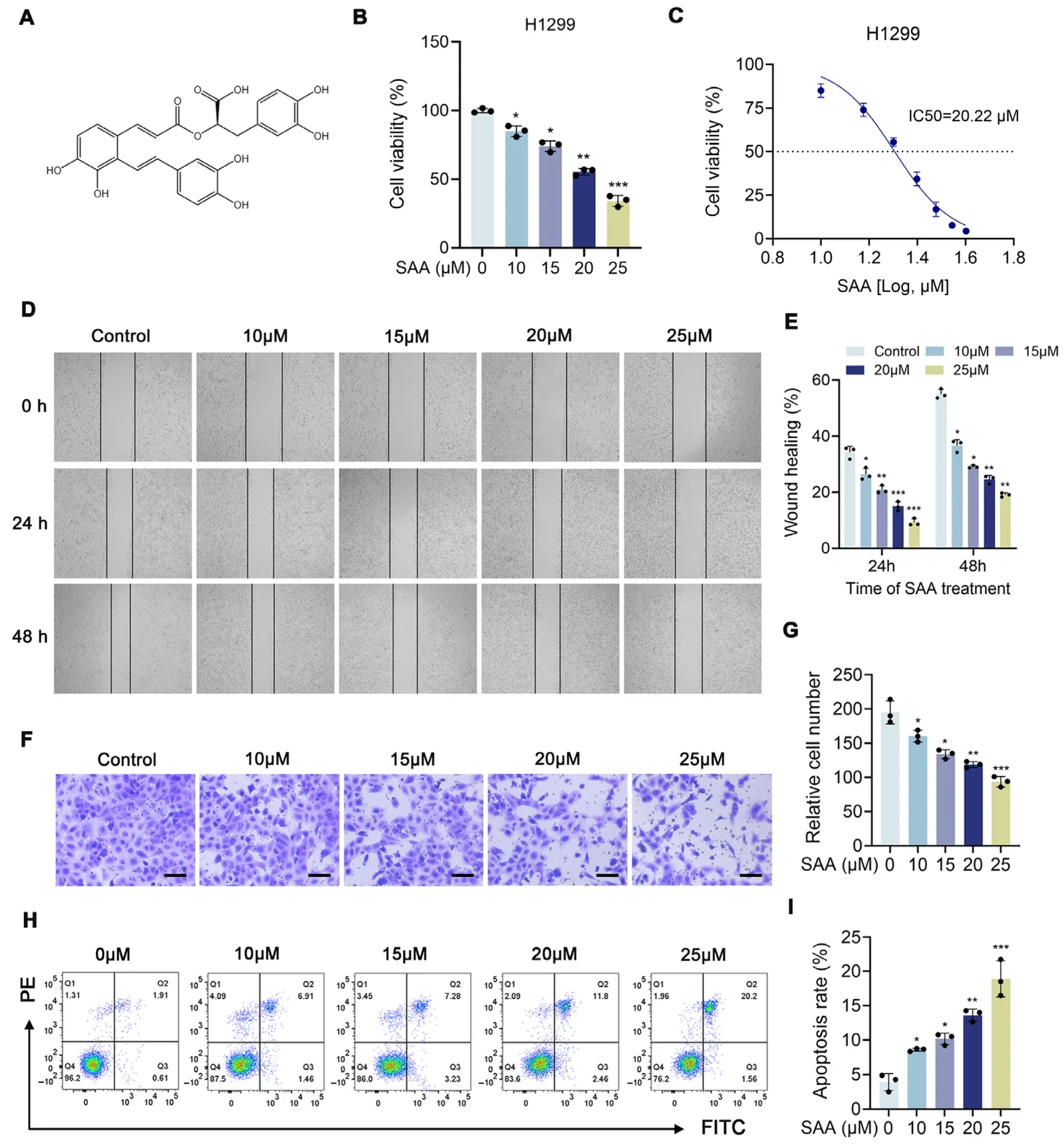
SAA suppresses proliferation and induces apoptosis of H1299 cells in vitro. (**A**) Chemical structure of SAA. (**B**,**C**) Cell viability of H1299 cells treated with SAA (0, 10, 15, 20 and 25 μM) for 24 h was assessed by CCK-8 assay. (**D**,**E**) Wound healing changes in H1299 cells with different concentrations of SAA for 0, 24, and 48 h. (**F**,**G**) Transwell experiment to investigate the effect of SAA on H1299 cells. Scale bar: 100 μm. (**H**,**I**) Cell apoptosis of H1299 cells assessed via flow cytometry. Data are presented as mean ± SD. Quantitative analyses were derived from at least three independent experiments. Statistical comparisons were performed using one-way ANOVA followed by Tukey’s multiple-comparison post hoc test. * *p* < 0.05, ** *p* < 0.01, *** *p* < 0.001.

The CCK-8 assay was used to assess the cytotoxic effects of SAA on H1299 cells. H1299 cells were treated with different concentrations of SAA for 24 h, and the results showed that SAA significantly inhibited the viability of H1299 cells (Figure 1B). The IC50 value for H1299 cells after 24 h was 20.22 μM (Figure 1C). Subsequently, wound healing assays were performed to determine whether SAA influenced cell migration. The results indicated that H1299 cells in the control group exhibited obvious wound closure during the observation period, whereas SAA treatment slowed wound closure (Figure 1D,E). Transwell-based invasion assays revealed that the number of cells that migrated through the membrane decreased progressively as SAA concentrations increased (Figure 1F,G). Finally, flow cytometry analysis showed that SAA induced apoptosis in H1299 cells (Figure 1H,I). In summary, these results indicate that SAA exhibits potent antitumor activity against NSCLC cells, supporting its therapeutic potential in the treatment of NSCLC.

### 3.2. SAA Inhibits NSCLC Cell Growth In Vivo

**Figure 2:**
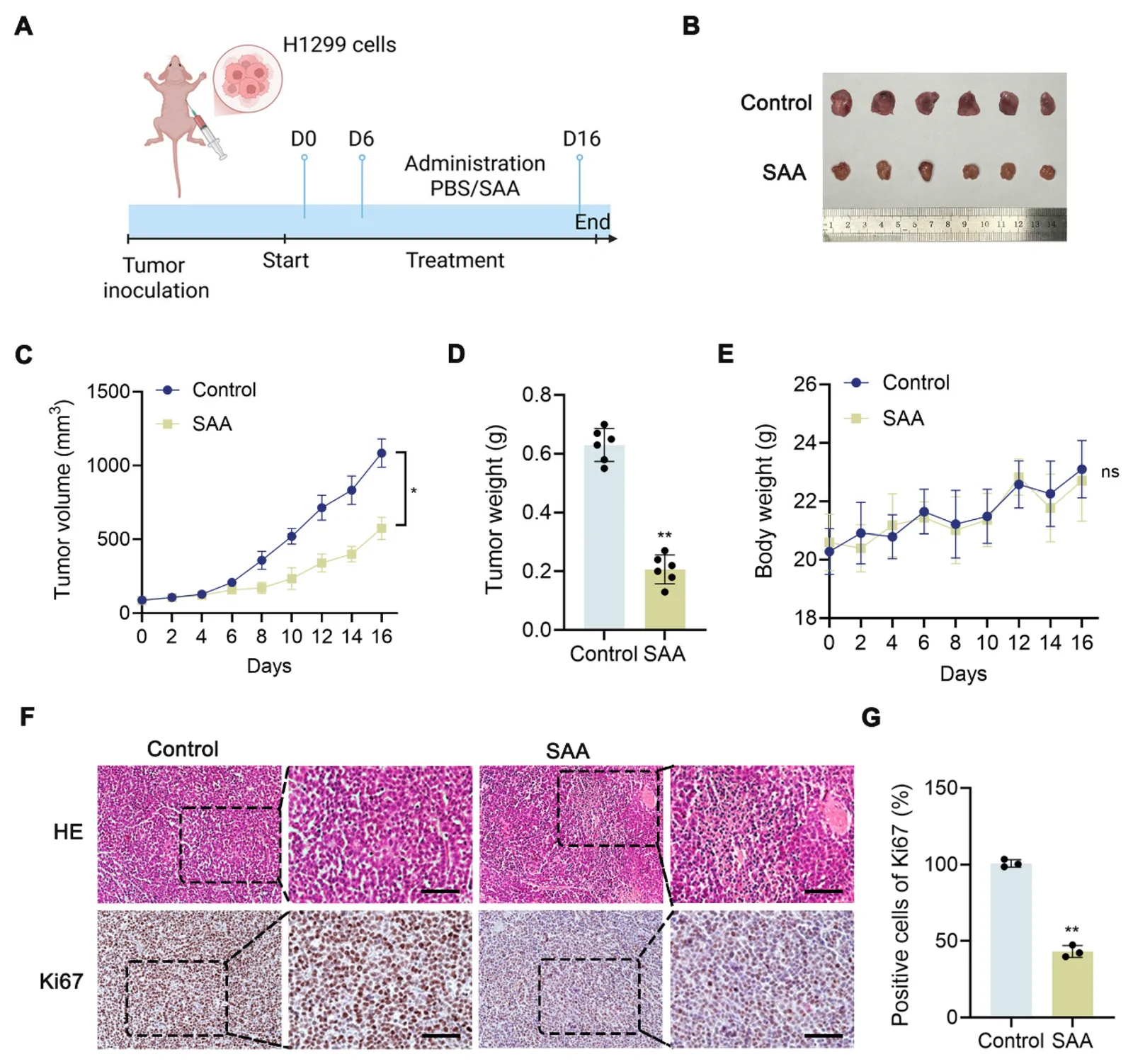
The effect of SAA on the in vivo growth of NSCLC cells. (**A**) Schematic diagram of the in vivo experiment on the administration of SAA. (**B**) Photograph of excised NSCLC tumor tissues. (**C**,**D**) Analysis of tumor volume changes during treatment and endpoint tumor weight. (**E**) Monitoring changes in the body weight of nude mice. (**F**,**G**) HE and IHC staining. Scale bar: 50 μm. Data are presented as mean ± SD. Each group included six mice. Tumor volume changes over time were analyzed using repeated-measures ANOVA. Endpoint tumor weight and body weight comparisons were performed using two-tailed Student’s *t*-test. * *p* < 0.05, ** *p* < 0.01.

Encouraged by the results of in vitro studies, we conducted further investigations into the effects of SAA on NSCLC under in vivo conditions (Figure 2A). Tumor volumes were measured every 2 days. The growth curve showed a significant reduction in tumor volume (Figure 2C), and the tumor weight in the SAA-treated group was substantially lower compared with the control group (Figure 2B,D). It is worth noting that there was no significant difference in body weight between the SAA-treated group and the control group (Figure 2E). Compared to the control group, HE staining revealed marked partial necrosis of tumor cells in the SAA-treated group, with distinct vacuoles present in the tumor tissue (Figure 2F). In addition, IHC analysis showed that SAA reduced Ki67 expression in tumor tissue (Figure 2F,G). In summary, these results indicate that SAA exhibits anti-NSCLC activity in vivo.

### 3.3. Metabolomics Analyses Based on MSI

**Figure 3:**
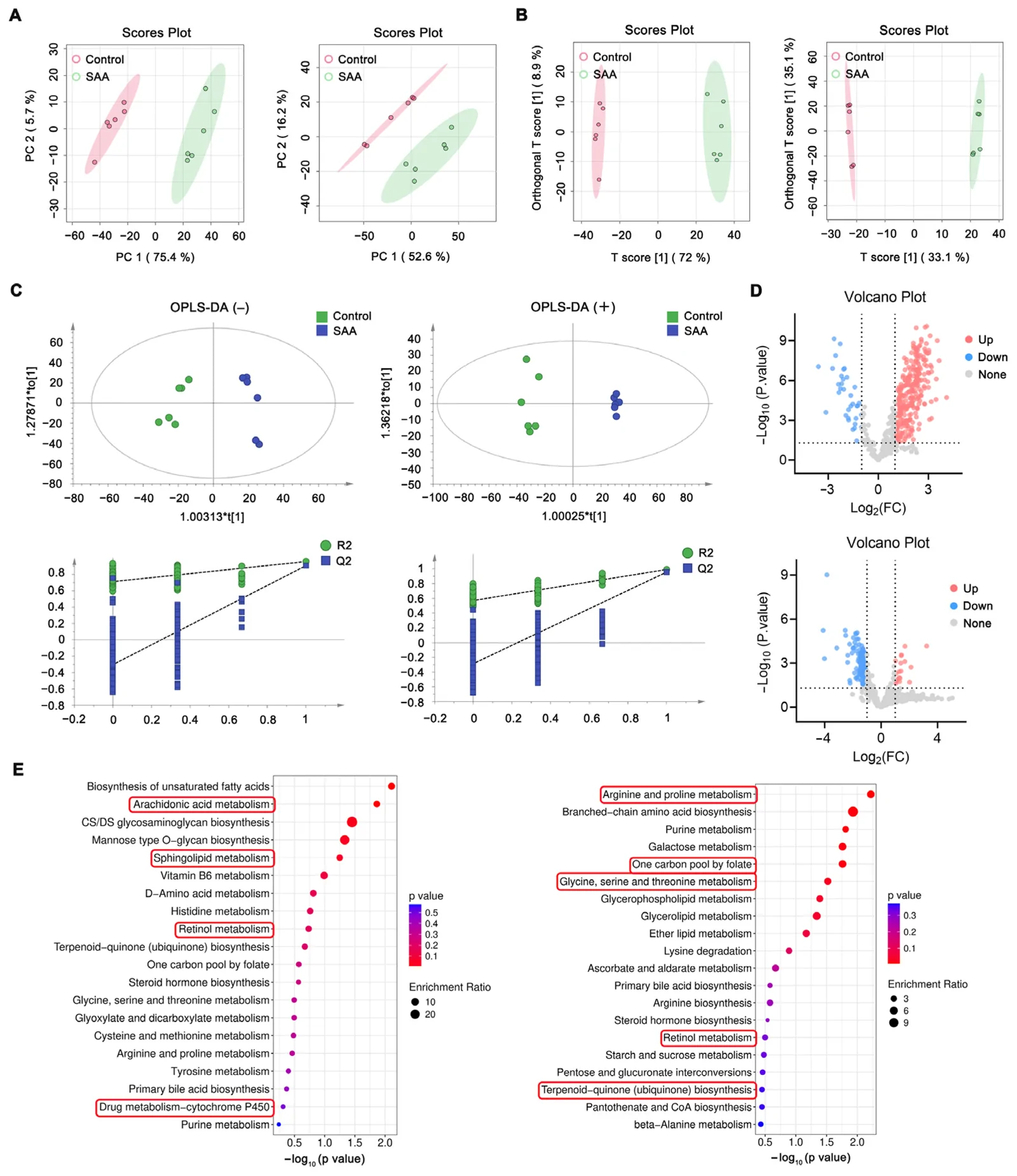
Metabolic changes in tumor tissue sections after treatment with SAA using DESI-MSI. (**A**) PCA score plots showing the metabolic differences between control and SAA groups. Negative ions left; positive ions right. (**B**) OPLS-DA score plots. Negative ions left; positive ions right. (**C**) The OPLS-DA score plots and permutations test in positive and negative ion modes. The asterisk (*) indicates statistical significance of the corresponding component. (**D**) Volcano plots. Upregulated metabolic features are indicated by red dots, whereas blue dots signify downregulated ones. Negative ions above; positive ions below. (**E**) KEGG pathway analysis. Negative ions left; positive ions right.

To investigate the metabolic alterations induced by SAA treatment in tumor tissues, DESI-MSI-based spatial metabolomics analysis was performed. Principal component analysis (PCA) was initially conducted to evaluate the overall metabolic differences between the control and SAA-treated groups. The PCA score plots showed clear separation between the two groups in both negative- and positive-ion modes, indicating distinct metabolic profiles following SAA intervention (Figure 3A). To further evaluate the group discrimination and robustness of the multivariate model, orthogonal partial least squares discriminant analysis (OPLS-DA) was performed. The OPLS-DA score plots demonstrated clear separation between the control and SAA-treated groups in both ion modes (Figure 3B). Permutation tests were subsequently conducted to evaluate model reliability and assess potential overfitting, supporting the stability and predictive performance of the OPLS-DA models (Figure 3C). As shown in Figure 3D, volcano plots were generated for both positive and negative ion modes. The volcano plot results indicate that, in the negative ion mode, 312 differential metabolic features were identified, of which 278 were upregulated and 34 were downregulated in the SAA-treated group relative to the control group. In the positive ion mode, 154 differential metabolic features were identified, including 20 upregulated and 134 downregulated features. The identified differential metabolic features were further subjected to KEGG pathway enrichment analysis using MetaboAnalyst, and the top 20 enriched pathways were visualized (Figure 3E). Among these, potential pathways related to negative ions include arachidonic acid (AA) metabolism, sphingolipid metabolism, Retinol metabolism, and drug metabolism–cytochrome P450 (Figure 3E). Potential pathways in cations include Arginine and proline metabolism, one-carbon pool by folate, Glycine, serine and threonine metabolism, Retinol metabolism, and Terpenoid-quinone (ubiquinone) biosynthesis (Table S2).

### 3.4. DESI-MSI Reveals SAA-Associated Spatial Metabolic Alterations in Tumor Tissues

To acquire a more comprehensive understanding of metabolic alterations and their spatial distribution following SAA treatment, we further conducted DESI-MSI analyses on tumor sections taken from mice in both the control and SAA groups. Differential metabolic features meeting the criteria defined in Section 2.7 were selected for subsequent spatial visualization. For quantitative MSI intensity comparisons, six independent tumors from six mice were analyzed per group, with one tissue section and one tumor ROI analyzed from each tumor. TIC-normalized ROI-level intensities were used for statistical comparisons, and individual MSI pixels or scans were not treated as independent biological replicates. Ultimately, 10 selected DESI-MSI features were retained for spatial visualization and quantitative comparison (Table S3).

**Figure 4:**
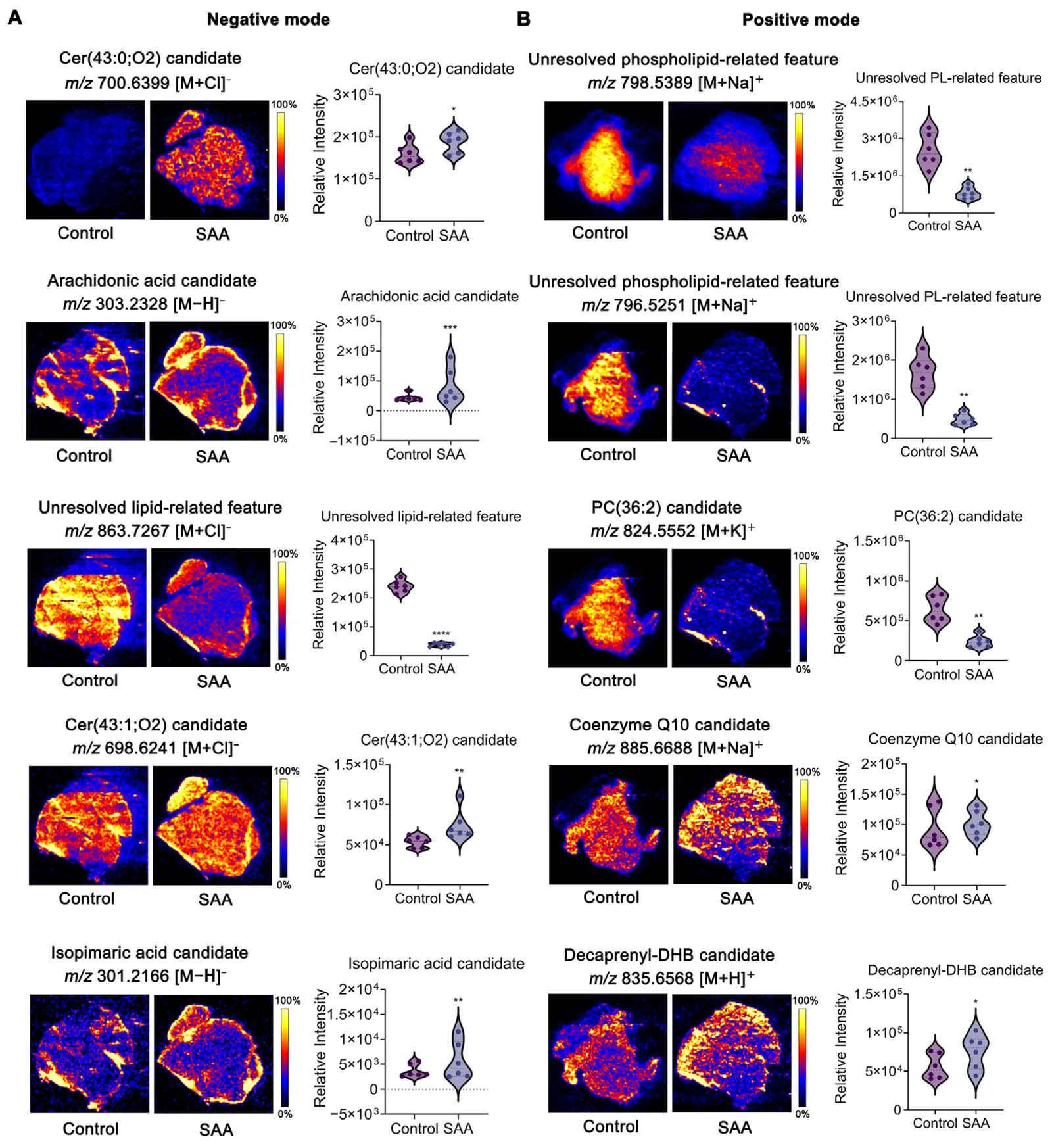
Spatial distribution of selected metabolic features in tumor tissue. (**A**,**B**) Representative DESI-MSI ion images of selected metabolic features. The highest metabolite signal intensity is represented by yellow, while the lowest is denoted by blue. Data are presented as mean ± SD. Data were obtained from six independent tumor samples per group; *n* = 6 biological replicates per group. Statistical comparisons were performed using two-tailed Student’s *t*-test. * *p* < 0.05, ** *p* < 0.01, *** *p* < 0.001, **** *p* < 0.0001 vs. control.

As shown in Figure 4, these selected features included ceramide candidates, phospholipid-related features, an arachidonic acid candidate, and lipid- and ubiquinone-related features. There was a significant upward trend in ceramide levels in the SAA group, such as the Cer(43:0;O2) candidate and the Cer(43:1;O2) candidate. Ceramides have been reported to be key pro-apoptotic sphingolipids capable of mediating the growth inhibition of tumor cells [17]. Therefore, the observed changes in these ceramide-related features may represent metabolic alterations associated with the SAA-induced apoptotic phenotype, although their direct causal contribution to apoptosis was not established in the present study. SAA treatment significantly reduced the levels of two unresolved phospholipid-related features (*m*/*z* 798.5389 and *m*/*z* 796.5251) and the PC(36:2) candidate. Glycerophospholipids are compounds capable of regulating carcinogenic cascades [18,19], and PC is a major membrane phospholipid involved in maintaining membrane organization and cellular signaling [20]. Accordingly, the observed phospholipid-related alterations suggest that SAA treatment is accompanied by remodeling of tumor lipid metabolism. These coordinated changes may therefore reflect perturbation of membrane lipid homeostasis associated with the antitumor response to SAA. In parallel, the unresolved lipid-related feature at *m*/*z* 863.7267 was markedly reduced following SAA treatment, further supporting broader remodeling of tumor lipid metabolism, although its precise structural identity remains unresolved.

Another key finding was that SAA treatment significantly increased the intensity of the arachidonic acid candidate, whereas the coenzyme Q10 and Decaprenyl-DHB candidates exhibited treatment-associated spatial and intensity alterations. As a key polyunsaturated fatty acid, AA metabolism has complex and context-dependent roles in tumor progression. On the one hand, its downstream metabolites can promote tumor cell proliferation, angiogenesis, and metastasis. On the other hand, changes in intracellular free AA may accompany membrane phospholipid turnover and oxidative stress-associated lipid remodeling [21,22]. Taken together with the alterations in phospholipid- and ubiquinone-related features, the increased intensity of the arachidonic acid candidate suggests that SAA treatment may disturb lipid and redox homeostasis within tumor tissues. These metabolic findings are biologically consistent with the independently observed increase in ROS and mitochondrial dysfunction, although they do not establish that individual MSI-detected features directly cause these cellular phenotypes.

In addition, SAA treatment caused significant changes in the spatial intensity of the isopimaric acid candidate. Recent studies have shown that isopimaric acid can induce apoptosis while causing mitochondrial dysfunction and the accumulation of ROS [23]. Although this observation may provide an additional metabolic clue relevant to SAA-induced oxidative stress and apoptosis, the putative annotation requires further structural confirmation before a compound-specific biological role can be assigned.

Collectively, the spatial alterations in these metabolic features support a potential association between SAA-induced metabolic remodeling, altered redox homeostasis, and mitochondrial apoptosis, while the causal contribution of individual MSI-detected features remains to be established.

### 3.5. Network Pharmacology Analysis

DESI-MSI results revealed significant differences in SAA-induced sphingolipid metabolism, arachidonic acid metabolism, and redox homeostasis, suggesting potential remodeling of lipid and redox homeostasis associated with the antitumor response to SAA. To further explore the potential molecular targets and signaling pathways associated with these effects, we subsequently performed network pharmacology-based target prediction and pathway enrichment analysis.

**Figure 5:**
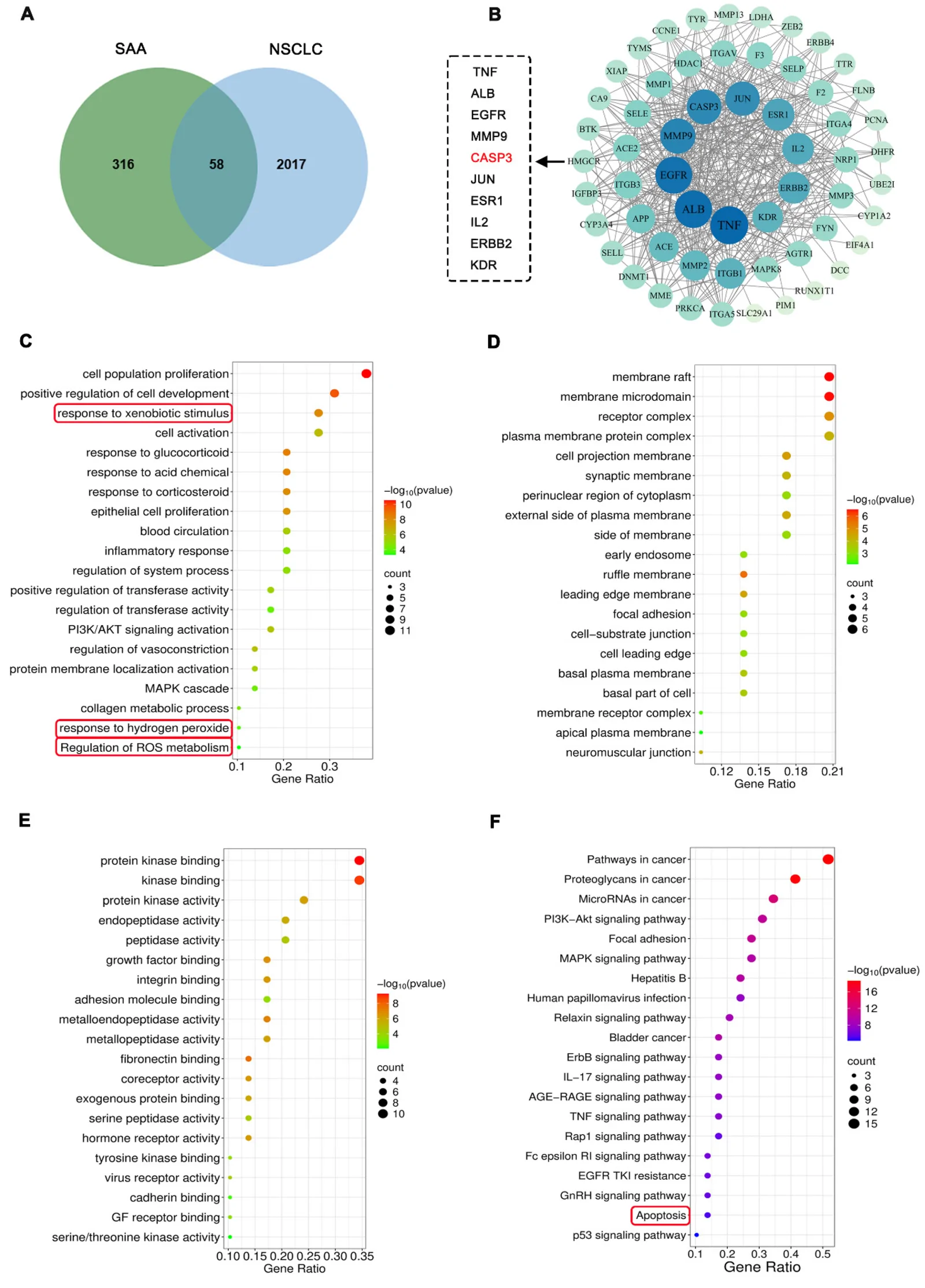
Network pharmacology analysis of potential SAA targets in NSCLC. (**A**) Venn diagram of 58 intersection targets. (**B**) PPI network. (**C**–**F**) GO and KEGG enrichment analysis of intersection targets.

We retrieved 316 SAA targets from databases, including SwissTargetPrediction and PharmMapper. We then utilized three databases including GeneCards, DrugBank, and OMIM for NSCLC. After data merging and duplicate removal, we identified 58 common targets (Figure 5A). We performed PPI analysis on the 58 targets associated with SAA-NSCLC that we identified (Figure 5B). Based on the degree values, the top 10 core target proteins were TNF, ALB, EGFR, MMP9, CASP3, JUN, ESR1, IL2, ERBB2, and KDR. Enrichment analysis was performed based on these intersection targets. The GO analysis results, shown in Figure 5C–E, highlight two biological processes: cellular responses to ROS and oxidative stress responses. KEGG results suggested that SAA may exert its therapeutic effects by regulating key signaling pathways such as PI3K-Akt, MAPK, apoptosis, and p53 (Figure 5F). It is worth noting that these related targets and signaling pathways are all associated with reactive oxygen species and apoptosis, consistent with the findings from MSI. This suggests that SAA may exert its anticancer effects by regulating caspase-related pathways through the accumulation of arachidonic acid and the depletion of antioxidants.

### 3.6. Molecular Docking Analysis

**Figure 6:**
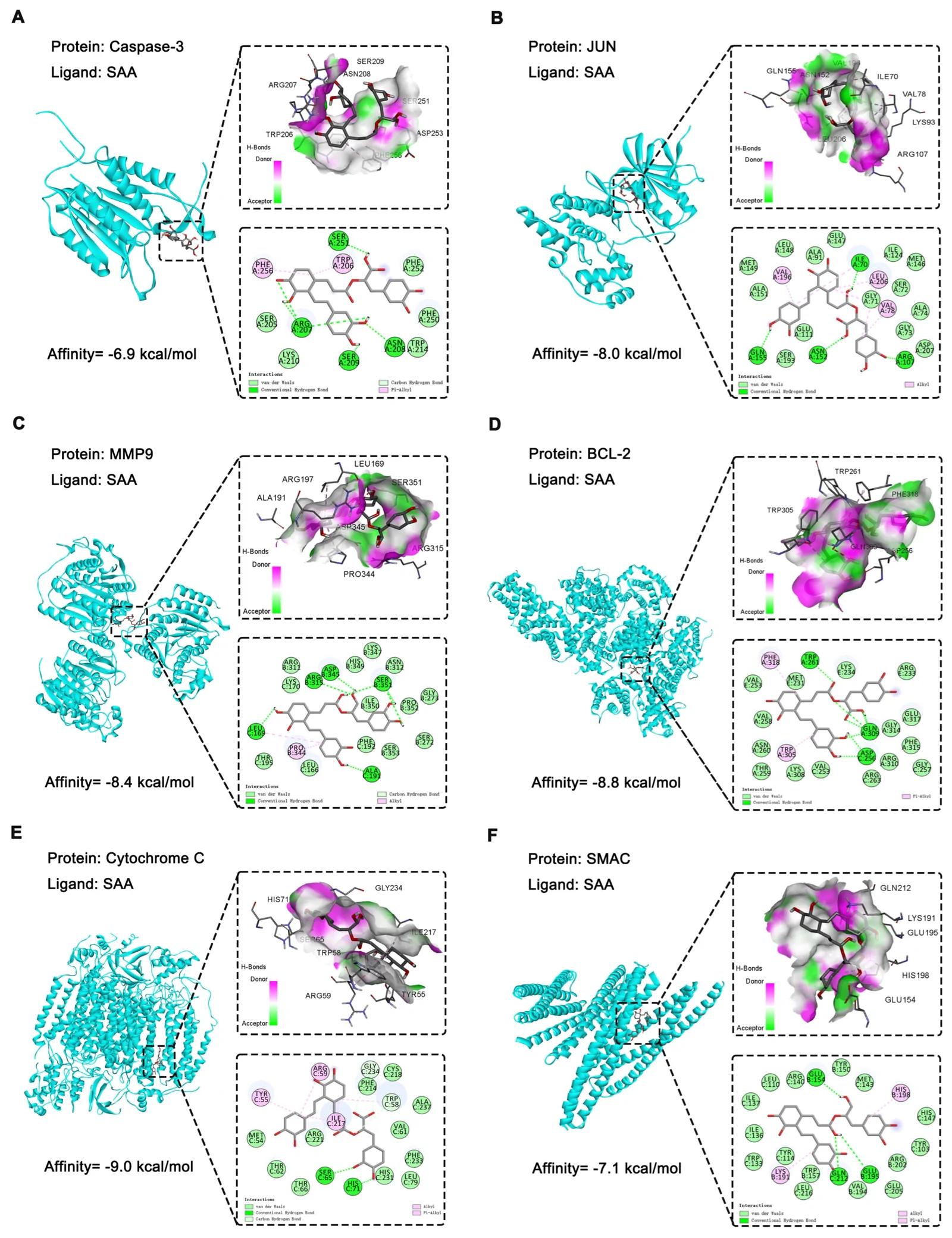
Molecular docking of SAA with the targets. (**A**) Caspase-3. (**B**) JUN. (**C**) MMP9. (**D**) BCL-2. (**E**) Cytochrome C. (**F**) SMAC.

Based on the foregoing analytical findings, it is suggested that the anti-NSCLC effects of SAA may be closely related to apoptosis- and oxidative stress-related mechanisms. Molecular docking techniques were used to investigate the binding affinities of three NSCLC-related targets (Caspase-3, JUN, and MMP9) and three oxidative stress-related targets (BCL-2, Cytochrome C, and SMAC) (Figure 6A–F). To evaluate the performance of the docking protocol, the native co-crystallized ligands of the applicable protein structures were redocked into their corresponding binding sites before docking with SAA. The RMSD values between the crystallographic and redocked ligand poses were 0.906 Å for Caspase-3, 1.166 Å for JUN, 1.48 Å for MMP9, 0.173 Å for BCL-2, 0.056 Å for Cytochrome c, and 1.546 Å for SMAC. All RMSD values were below 2.0 Å, indicating good precision and a good binding pattern. Notably, the predicted docking scores of SAA with the six targets were all below −5.0 kcal/mol, suggesting energetically favorable predicted interactions between SAA and the selected targets. In particular, the binding energies to BCL-2 (−8.8 kcal/mol) and Cytochrome C (−9.0 kcal/mol) were significantly lower than the commonly recognized strong binding threshold of −7.0 kcal/mol [24]. These molecular docking results provide supportive computational evidence for the potential interactions between SAA and the selected targets and complement the network pharmacology and DESI-MSI findings.

### 3.7. SAA Induces Oxidative Stress-Related Mitochondrial Dysfunction, Which Contributes to Apoptosis in H1299 Cells

**Figure 7:**
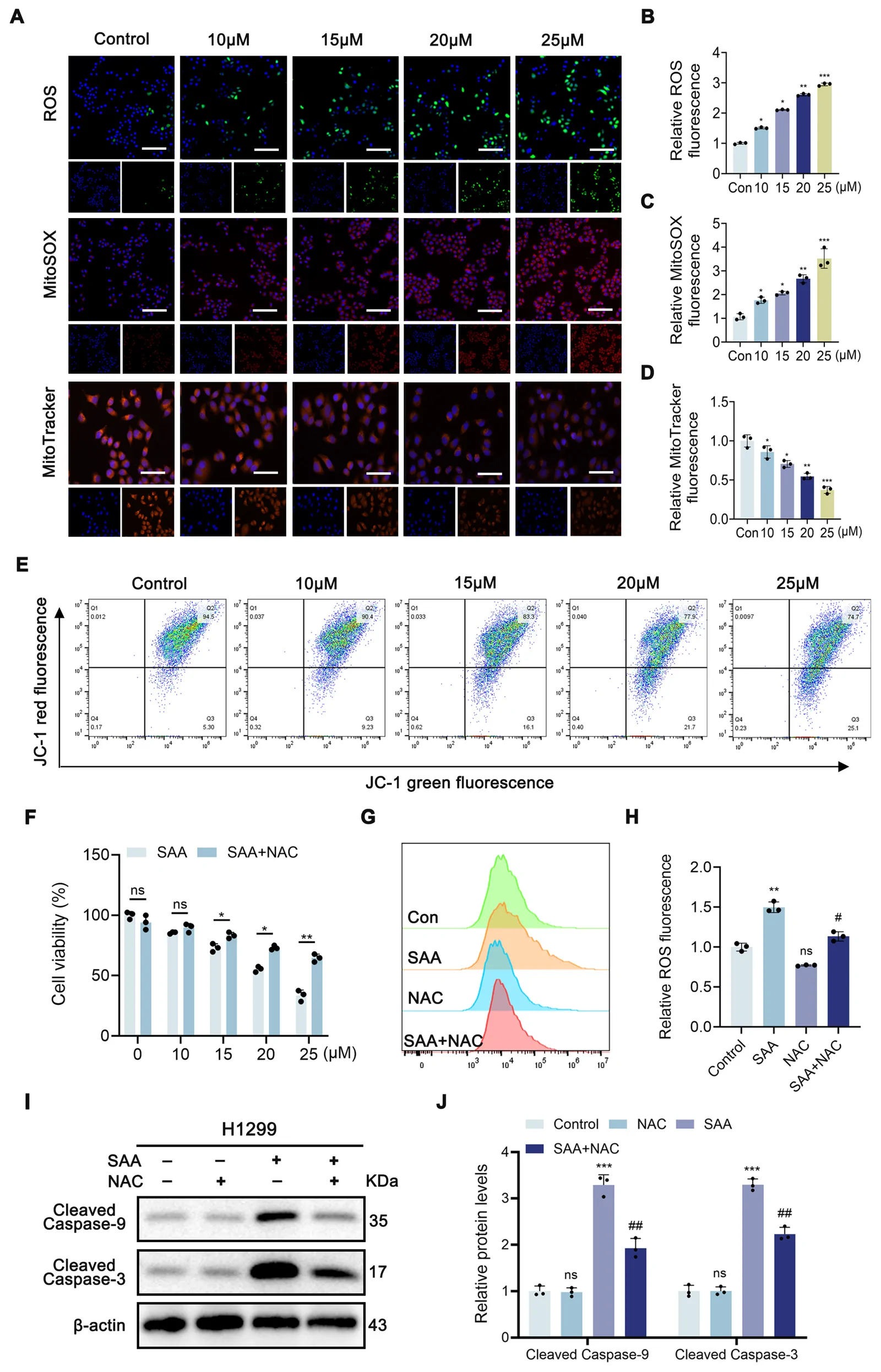
SAA induces apoptosis in H1299 cells by increasing ROS production and causing mitochondrial damage. (**A**) Representative fluorescence images of intracellular ROS, MitoSOX, and MitoTracker staining in H1299 cells treated with SAA (0, 10, 15, 20 and 25 μM). Scale bar: 100 μm. (**B**) Quantification of intracellular ROS levels in SAA-treated H1299 cells. (**C**) Quantification of MitoSOX fluorescence in SAA-treated H1299 cells. (**D**) Quantification of MitoTracker fluorescence in SAA-treated H1299 cells. (**E**) Determination of the distribution ratio of JC-1 red and JC-1 green cell populations in SAA-treated H1299 cells (0, 10, 15, 20 and 25 μM) by flow cytometry. (**F**) H1299 cells were treated with SAA (25 μM) with or without NAC (2.5 mM) for 24 h. (**G**,**H**) Intracellular ROS levels in H1299 cells treated with SAA (25 μM) for 24 h, in the presence or absence of NAC (2.5 mM). (**I**,**J**) Effects of NAC (2.5 mM) on SAA-induced changes in cleaved Caspase-9 and cleaved Caspase-3 protein detected by Western blotting. Data are presented as mean ± SD. Quantitative analyses were derived from at least three independent experiments. Statistical comparisons were performed using one-way ANOVA followed by Tukey’s multiple-comparison post hoc test. * *p* < 0.05, ** *p* < 0.01, *** *p* < 0.001 vs. control and # *p* < 0.05, ## *p* < 0.01 vs. SAA (25 μM). Original images of Figure 7I can be found in Supplementary Materials.

The DESI-MSI results revealed treatment-associated alterations in antioxidant-related metabolic features within tumor tissues, while network pharmacology and molecular docking suggested the potential involvement of apoptosis- and oxidative stress-related targets. Our results indicated that SAA significantly increased intracellular ROS levels in H1299 cells (Figure 7A,B). Given the observed increase in intracellular ROS, we further investigated whether mitochondrial ROS was also increased following SAA treatment. To assess this, we performed staining with MitoSOX, a mitochondrial ROS marker, and found that following SAA treatment, the MitoSOX fluorescence intensity in H1299 cells increased in a concentration-dependent manner (Figure 7A,C). Because excessive mitochondrial ROS is closely associated with mitochondrial dysfunction, we next evaluated mitochondrial status and membrane potential. MitoTracker fluorescence progressively decreased with increasing SAA concentrations (Figure 7A,D), while JC-1 analysis showed a concentration-dependent loss of mitochondrial membrane potential (Figure 7E). Together, these findings indicate that SAA treatment is accompanied by mitochondrial oxidative stress and mitochondrial dysfunction. Subsequently, intervention with the ROS scavenger N-acetylcysteine (NAC) showed that NAC treatment significantly reversed SAA-induced reduction in cell viability (Figure 7F). Similarly, the SAA-induced increase in ROS levels was partially reversed by NAC (Figure 7G,H). In addition, NAC treatment significantly reversed SAA-induced protein expression (Figure 7I,J), further supporting the activation of the mitochondrial caspase-related apoptotic cascade. These results functionally support the contribution of ROS accumulation to SAA-induced mitochondrial apoptosis, while the MSI-detected metabolic alterations may represent associated metabolic responses during this process.

### 3.8. SAA Induces Caspase-Dependent Apoptosis in H1299 Cells

**Figure 8:**
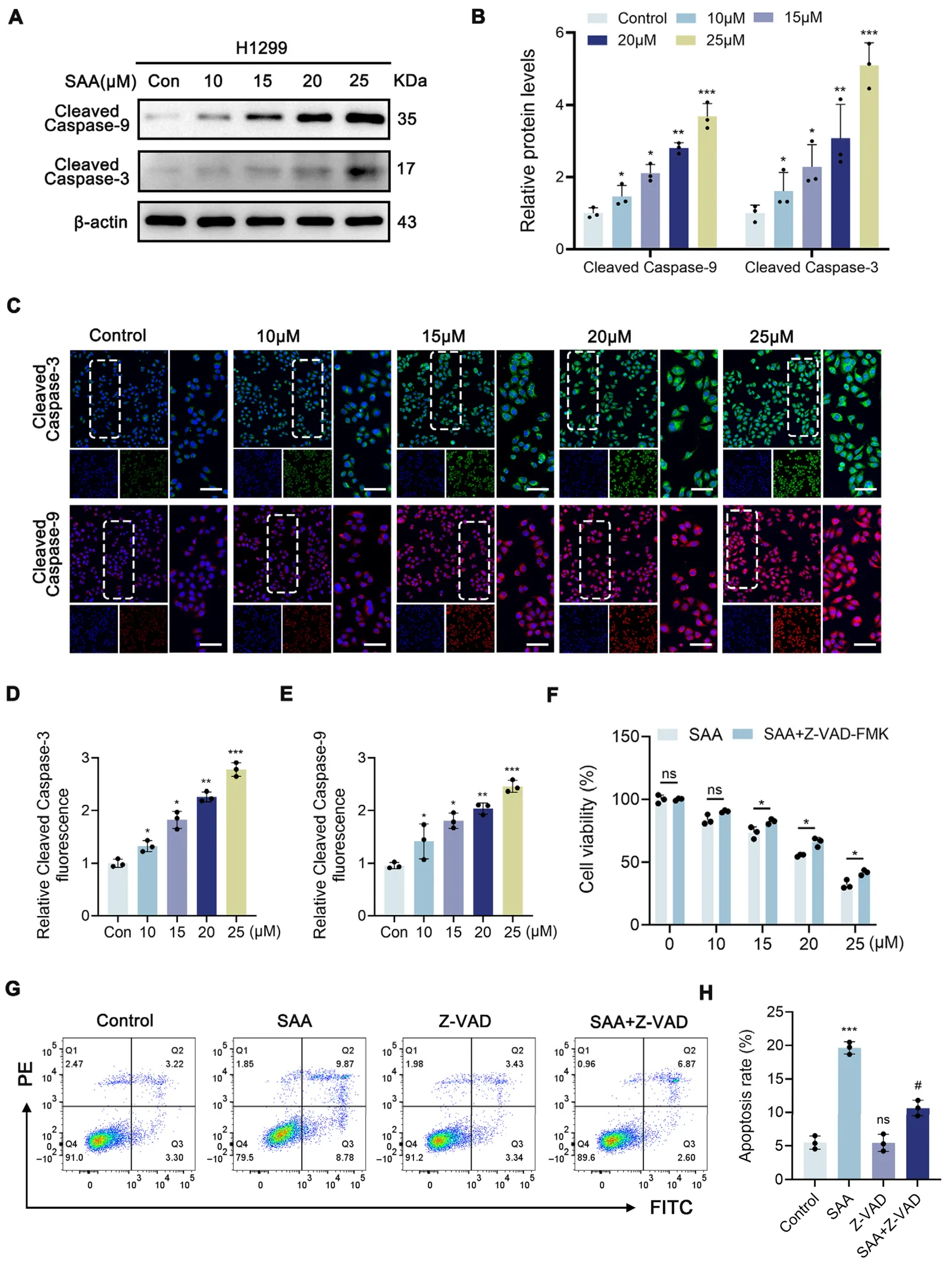
SAA promotes apoptosis in H1299 cells via the caspase-dependent pathway. (**A**,**B**) Western blot assessment of cleaved Caspase-9 and cleaved Caspase-3 levels in H1299 cells following exposure to SAA. (**C**–**E**) Cleaved Caspase-3 and cleaved Caspase-9 protein signals detected by IF assays. Scale bar: 100 μm. (**F**) H1299 cells were treated with SAA (0, 10, 15, 20 and 25 μM) with or without Z-VAD (20 μM) for 24 h. (**G**,**H**) Apoptosis of H1299 cells assessed via flow cytometry, following 24 h exposure to SAA (25 μM) with or without Z-VAD (20 μM). Data are presented as mean ± SD. Quantitative analyses were derived from at least three independent experiments. Statistical comparisons were performed using one-way ANOVA followed by Tukey’s multiple-comparison post hoc test. * *p* < 0.05, ** *p* < 0.01, *** *p* < 0.001 vs. control and # *p* < 0.05 vs. SAA (25 μM). Original images of Figure 8A can be found in Supplementary Materials.

To determine whether SAA activates the caspase cascade in vivo, we performed IHC staining of tumor tissues from the xenograft model. IHC analysis revealed that significantly elevated levels of cleaved caspase-3 and -9 were observed in tumor sections from SAA-treated mice relative to the control group (Supplementary Figure S1A–C). Consistently, Western blot analysis showed that SAA treatment concentration dependently increased the levels of cleaved caspase-9 and cleaved caspase-3 in H1299 cells in vitro (Figure 8A,B). In addition, immunofluorescence staining analysis demonstrated that the fluorescence intensities of cleaved caspase-9 and cleaved caspase-3 were markedly increased in SAA-treated H1299 cells (Figure 8C–E). In addition, to further investigate whether apoptosis contributes to the SAA-induced cell death process, the apoptosis inhibitor Z-VAD-FMK (Z-VAD) was employed in co-culture experiments. Notably, the results revealed that Z-VAD could block the cell death induced by SAA treatment (Figure 8F). Additionally, Z-VAD treatment markedly suppressed SAA-induced apoptosis in H1299 cells (Figure 8G,H). Together, these results demonstrate that SAA triggers caspase-mediated apoptosis both in vitro and in vivo, supporting the apoptosis-related mechanisms suggested by the integrated analyses of DESI-MSI, network pharmacology, and molecular docking. This caspase-centric execution mechanism provides functional evidence for SAA-induced cell death and complements the spatial metabolic alterations observed in tumor tissues.

## 4. Discussion

This study investigated the potential molecular mechanism by which SAA inhibits NSCLC through the integration of MSI-based spatial metabolomics, network pharmacology, and molecular docking. Our DESI-MSI analysis revealed that SAA treatment induced spatially resolved metabolic reprogramming within tumor tissues, characterized by significant accumulation of ceramides and arachidonic acid-associated metabolic alterations, alongside pronounced depletion of endogenous antioxidants including coenzyme Q10 and Decaprenyl-DHB candidates. These metabolic alterations were accompanied by findings from network pharmacology and molecular docking analyses, which suggested the potential involvement of apoptosis- and oxidative stress-related pathways in SAA’s anti-NSCLC effects. Specifically, SAA treatment significantly increased intracellular ROS and mitochondrial superoxide-associated signals, which was associated with caspase cascade activation and reduced cell proliferation. ROS scavengers could also reverse the SAA-induced caspase signaling. To our knowledge, this is the first study to use metabolomics and MSI to reveal the potential mechanisms underlying SAA’s action against NSCLC.

Studies have shown that the production of ROS within cells can lead to mitochondrial dysfunction, which in turn triggers apoptosis [25,26]. The role of ROS in promoting cell cycle arrest in various cancers has been well established; therefore, ROS is taken into account in the development of many anticancer drugs [27,28]. Although previous studies have confirmed that SAA exhibited antitumor activity in various cancers, its specific regulatory mechanism regarding mitochondrial metabolism in NSCLC remains unclear. We observed that SAA treatment significantly increased intracellular ROS levels, while MitoSOX analysis further suggested that mitochondrial ROS may contribute to the observed oxidative response. These changes were accompanied by activation of the caspase cascade and inhibition of cell proliferation. ROS scavengers could also reverse SAA-induced caspase signaling, supporting the involvement of ROS accumulation in SAA-mediated apoptotic responses. Together with our previous findings, these results suggest that oxidative stress may represent a shared upstream response to SAA, whereas distinct downstream regulated cell-death programs, including ferroptosis and mitochondrial apoptosis, may be engaged depending on the cellular context. However, the present data do not establish direct mechanistic crosstalk between ferroptosis and apoptosis, and this relationship warrants further investigation.

Beyond these phenotypic observations, our MSI data provided spatially resolved metabolic evidence that substantially deepens the mechanistic understanding. We found that following SAA intervention, there was a significant accumulation of ceramides (such as Cer(43:0;O2) and Cer(43:1;O2) candidates) and an AA candidate, along with a marked depletion of antioxidants, including coenzyme Q10 and Decaprenyl-DHB. Ceramides are bioactive sphingolipids known to participate in the regulation of cell death processes [29], suggesting that the observed changes in ceramide-related features may represent one metabolic alteration associated with the pro-apoptotic response induced by SAA. AA and its metabolites may influence tumor cell proliferation and survival through multiple metabolic pathways [30]. The observed alterations in the arachidonic acid candidate and ceramide-related features suggest that lipid metabolic remodeling may accompany the apoptotic response following SAA treatment. However, whether these metabolic changes directly regulate caspase activation requires further investigation. Furthermore, ROS are widely recognized as potent inducers of apoptosis, with the ability to stimulate the mitochondrial intrinsic pathway, the death receptor extrinsic pathway, and the endoplasmic reticulum stress pathway [31]. Combined with our previous observation that SAA significantly elevates intracellular ROS levels in NSCLC cells, the concurrent depletion of antioxidants observed in tumor tissues suggests that SAA may alter redox homeostasis, thereby contributing to oxidative stress-associated apoptotic signaling. The integration of network pharmacology and MSI provides a novel strategy for the assessment of pharmacodynamics and the exploration of mechanisms underlying multi-target drugs.

Despite these findings, several limitations warrant acknowledgment. First, although network pharmacology and molecular docking suggested a potential interaction between SAA and Caspase-3, the specific molecular interactions between SAA and the predicted targets, as well as the regulatory roles of key metabolic alterations in these pathways, require further experimental validation, for example, through gene knockout or overexpression approaches. Second, although DESI-MSI enabled visualization of treatment-associated spatial distribution patterns of metabolic features within tumor tissues, the present spatial metabolomics analysis was not co-registered with matched histopathological images, and metabolite distributions were not quantitatively evaluated within histologically defined regions of interest, such as viable and necrotic tumor regions. This limitation is particularly relevant because SAA treatment induced substantial tumor necrosis, and some of the observed spatial metabolic differences may therefore reflect changes in tissue composition or necrosis-associated metabolism rather than direct metabolic regulation by SAA. Future studies integrating DESI-MSI with HE-guided image registration and histopathology-defined ROI-specific analysis will be required to distinguish metabolic alterations occurring within viable tumor tissue from those associated with treatment-induced necrosis. Although DESI-MSI provided spatially resolved metabolic alterations associated with SAA treatment, the absence of authentic standards and MS/MS confirmation limits definitive structural identification of individual metabolites. Therefore, the detected ceramide-, arachidonic acid-, and coenzyme Q10-related features should be interpreted as associated metabolic changes rather than direct causal mediators of apoptosis. Moreover, although the present MSI findings revealed associations between metabolic alterations and the apoptotic phenotype, the causal relationships between specific metabolic changes and apoptotic execution remain to be established through further mechanistic studies. Although MitoSOX analysis provided evidence of increased mitochondrial superoxide-associated signals following SAA treatment, the precise causal contribution of mitochondrial ROS to downstream mitochondrial dysfunction and apoptotic signaling remains to be established. Future studies using mitochondria-targeted ROS scavengers will be required to further define this relationship. Finally, while the putatively annotated MSI-derived metabolic features may represent candidate metabolic signatures with potential translational value, their clinical relevance and predictive value remain to be established through validation in independent experimental models and patient cohorts. Future studies should therefore include larger clinical cohorts and targeted metabolomic analyses to determine the abundance, reproducibility, and clinical detectability of these metabolic alterations in serum and tumor tissues from patients with NSCLC and to evaluate their potential utility as predictive indicators of SAA response or prognostic monitoring. Collectively, these investigations will further refine the understanding of the potential mechanisms underlying the antitumor effects of SAA and provide a more rigorous basis for evaluating its future clinical translation.

In summary, this study is the first to utilize DESI-MSI-based spatial metabolomics, network pharmacology, and molecular docking in an integrated manner to perform spatially resolved metabolic profiling of SAA-induced apoptosis in NSCLC. We demonstrated that SAA treatment promotes apoptosis in NSCLC cells and that ROS accumulation is associated with mitochondria-dependent caspase cleavage. These findings reveal a potential mechanism underlying the anticancer effects of SAA and provide a new conceptual foundation for understanding SAA-induced metabolic remodeling and mitochondria-associated antitumor responses.

## 5. Conclusions

Taken together, this study found that SAA significantly inhibited the growth of NSCLC and provided insights into the potential mechanisms underlying SAA activity against NSCLC by integrating DESI-MSI-based spatial metabolomics, network pharmacology, and molecular docking. The spatial metabolic alterations induced by SAA in NSCLC were associated with changes in metabolites such as ceramides, glycerophospholipids, and arachidonic acid. Mechanistically, SAA appears to promote apoptosis in NSCLC cells through a ROS-associated mitochondrial dysfunction and caspase-dependent apoptotic pathway. These observations provide novel perspectives for understanding the potential antitumor activity of SAA in NSCLC.

## Data Availability

The original contributions presented in this study are included in the article/Supplementary Material. Further inquiries can be directed to the corresponding authors.

## Supplementary Materials

The following supporting information can be downloaded at https://www.mdpi.com/article/10.3390/biom16101434/s1: Figure S1: IHC staining of cleaved Caspase-3 and cleaved Caspase-9; Figure S2: Validation of the docking by redocking of co-crystallized ligands; Table S1: Validation parameters of OPLS-DA models in positive- and negative-ion modes; Table S2-1: Metabolic pathway information table for Control vs SAA Group in Negative ions; Table S2-2: Metabolic pathway information table for Control vs SAA Group in Positive ions; Table S3: Differential DESI-MSI features and original database-derived annotations in the control and SAA-treated groups; Table S4: Molecular docking parameters.

## Abbreviations

The following abbreviations are used in this manuscript:

AA	Arachidonic acid
CCK-8	Cell counting kit-8
DESI	Desorption electrospray ionization
DCFH-DA	2:7-Dichlorodi-hydrofluorescein diacetate
GO	Gene ontology
IF	Immunofluorescence
IHC	Immunohistochemistry
KEGG	Kyoto encyclopedia of genes and genomes
MSI	Mass spectrometry imaging
NSCLC	Non-small-cell lung cancer
NAC	N-acetylcysteine
OPLS-DA	Orthogonal partial least squares discriminant analysis
PCA	Principal component analysis
PC	Phosphatidylcholine
ROS	Reactive oxygen species
SAA	Salvianolic acid A
Z-VAD	Z-VAD-FMK
