# Supplementary material for: Spatial Metabolomics-Based Integrated Analysis Provides Evidence for the Mechanism of Salvianolic Acid A-Induced Mitochondrial Apoptosis in Non-Small Cell Lung Cancer via the ROS-Dependent Caspase Pathway
Source: Biomolecules. 2026 Oct 2;16(10):1434. doi: 10.3390/biom16101434 (PMC13702214; doi:10.3390/biom16101434)
Supplement: Supplementary file 1 [file biomolecules-16-01434-s001.zip › biomolecules-4552319-supplementary.pdf]

Supplementary Material

Spatial Metabolomics-Based Integrated Analysis Provides Evidence for the Mechanism of Salvianolic Acid A-Induced Mitochondrial Apoptosis in Non-Small Cell Lung Cancer via the ROS-Dependent Caspase Pathway

Ruyu Jiang [1,†], Hairong Xiang [1,†], Lushuang Xie [1], Linfeng Zhao [1], Dawei Zeng [1], Yue Zhang [1], Jiazhen Xie [1], Zhenxu Pan [2], Yanju Gong [1,*], Lan Yang [1,*]

Table S1 Validation parameters of OPLS-DA models in positive- and negative-ion modes.

Ion mode	R^2X(cum)	R^2Y(cum)	Q^2(cum)	Cross-validation method	Number of permutations
Negative	0.326	0.711	0.55	7-fold Venetian blinds	200
Positive	0.709	0.571	0.48	7-fold Venetian blinds	200

Table S2-1 Metabolic pathway information table for Control vs SAA Group in Negative ions.

Number	Metabolite pathway name	Total metabolites	number of hits	P-value	Impact value	-log（P）
1	Biosynthesis of unsaturated fatty acids	36	3	0.00782	0.427	2.107
2	Arachidonic acid metabolism	44	3	0.0137	0.521	1.863
3	CS/DS glycosaminoglycan biosynthesis	3	1	0.0352	0.0355	1.450
4	Mannose type O-glycan biosynthesis	4	1	0.0466	0.0474	1.324
5	Sphingolipid metabolism	33	2	0.0564	0.391	1.249
6	Vitamin B6 metabolism	9	1	0.102	0.107	0.991

7	D-Amino acid metabolism	14	1	0.154	0.166	0.812
8	Histidine metabolism	16	1	0.174	0.19	0.759
9	Retinol metabolism	17	1	0.184	0.201	0.735
10	Terpenoid-quinone (ubiquinone) biosynthesis	20	1	0.213	0.237	0.672
11	One carbon pool by folate	26	1	0.268	0.308	0.572
12	Steroid hormone biosynthesis	86	2	0.271	1.02	0.567
13	Glycine, serine and threonine metabolism	32	1	0.32	0.379	0.495
14	Glyoxylate and dicarboxylate metabolism	32	1	0.32	0.379	0.495
15	Cysteine and methionine metabolism	33	1	0.328	0.391	0.484
16	Arginine and proline metabolism	35	1	0.344	0.415	0.463
17	Tyrosine metabolism	42	1	0.398	0.498	0.400
18	Primary bile acid biosynthesis	46	1	0.427	0.545	0.370
19	Drug metabolism - cytochrome P450	55	1	0.487	0.652	0.312
20	Purine metabolism	70	1	0.574	0.829	0.241

Table S2-2 Metabolic pathway information table for Control vs SAA Group in Positive ions

Number	Metabolite pathway name	Total metabolites	number of hits	P-value	Impact value	-log（P）
1	Arginine and proline metabolism	35	4	0.00602	0.76	2.22
2	Branched-chain amino acid biosynthesis	8	2	0.0118	0.174	1.93
3	Purine metabolism	70	5	0.0156	1.52	1.81
4	Galactose metabolism	4	1	0.0173	0.565	1.76

5	One carbon pool by folate	33	2	0.0173	0.565	1.76
6	Glycine, serine and threonine metabolism	9	1	0.0302	0.695	1.52
7	Glycerophospholipid metabolism	14	1	0.041	0.782	1.39
8	Glycerolipid metabolism	16	1	0.0454	0.348	1.34
9	Ether lipid metabolism	17	1	0.0682	0.434	1.17
10	Lysine degradation	20	1	0.129	0.63	0.89
11	Ascorbate and aldarate metabolism	26	1	0.215	0.239	0.67
12	Primary bile acid biosynthesis	86	2	0.264	0.999	0.58
13	Arginine biosynthesis	32	1	0.266	0.304	0.58
14	Steroid hormone biosynthesis	32	1	0.286	1.87	0.54
15	Retinol metabolism	33	1	0.313	0.369	0.50
16	Starch and sucrose metabolism	35	1	0.328	0.391	0.48
17	Pentose and glucuronate interconversions	42	1	0.343	0.413	0.46
18	Terpenoid-quinone (ubiquinone) biosynthesis	46	1	0.357	0.434	0.45
19	Pantothenate and CoA biosynthesis	55	1	0.357	0.434	0.45
20	beta-Alanine metabolism	70	1	0.371	0.456	0.43

Table S3 Differential DESI-MSI features and original database-derived annotations in the control and SAA-treated groups.

Original database annotation	Formula	Ion mode	Measured (m/z)	Error (ppm)	VIP	FC	P values	Confidence
Cer(43:0;O2) candidate	C43H87NO3	[M+Cl]−	700.6399	2.72	6.12	0.92	0.1578	Level 4

Arachidonic acid candidate	C20H32O2	[M−H]−	303.2328	0.46	5.81	1.86	0.1526	Level 4
Unresolved lipid-related feature	C54H100O5	[M+Cl]−	863.7267	0.26	3.24	6.28	0.0706	Level 5
Cer(43:1;O2) candidate	C43H85NO3	[M+Cl]−	698.6241	2.51	4.42	1.23	0.3196	Level 4
Isopimaric acid candidate	C20H30O2	[M−H]−	301.2166	2.29	1.70	1.53	0.0443	Level 4
Unresolved phospholipid-related feature	C45H78NO7P	[M+Na]+	798.5389	2.40	13.76	0.32	0.0135	Level 5
Unresolved phospholipid-related feature	C45H76NO7P	[M+Na]+	796.5251	0.07	12.03	0.29	0.0068	Level 5
PC(36:2) candidate	C44H84NO8P	[M+K]+	824.5552	1.71	5.88	0.37	0.0226	Level 4
Coenzyme Q10 candidate	C59H90O4	[M+Na]+	885.6688	4.89	2.51	1.89	0.0625	Level 4
Decaprenyl-dihydroxybenzoate candidate	C57H86O4	[M+H]+	835.6568	3.69	3.14	0.94	0.7468	Level 4

Note: Metabolite annotation confidence was assigned according to the five-level framework proposed for high-resolution mass spectrometry and adapted for mass spectrometry imaging. Level 4 denotes an unequivocal molecular formula supported by the available high-resolution MS information without unique structural confirmation, whereas Level 5 denotes an accurate-m/z feature for which the available data do not support an unequivocal molecular formula.

Table S4. Molecular docking parameters.

Protein	Crystal code	Coordinates (x, y, z)	Size (x, y, z)	Docking score (kcal/mol)	Number of runs	RMSD (Å)
Caspase-3	2J31	(26.549, 19.325, 58.505)	(47.25,47.25, 47.25)	-6.9	9	0.906
JUN	4Y46	(8.743, -20.126, -23.192)	(47.25,47.25, 47.25)	-8.0	9	1.166
MMP9	5I12	(19.057, -21.591,23.21)	(22.5, 22.5, 22.5)	-8.4	9	1.48
BCL-2	4HW3	(47.009, -24.189, 26.792)	(81.55, 81.55, 81.55)	-8.8	9	0.173
Cytochrome C	5Z62	(325.958, 329.91, 253.636)	(89.95, 89.95, 89.95)	-9.0	9	0.056
SMAC	6JX6	(-11.941, -40.164, 18.377)	(57.75, 57.75, 57.75)	-7.1	9	1.546

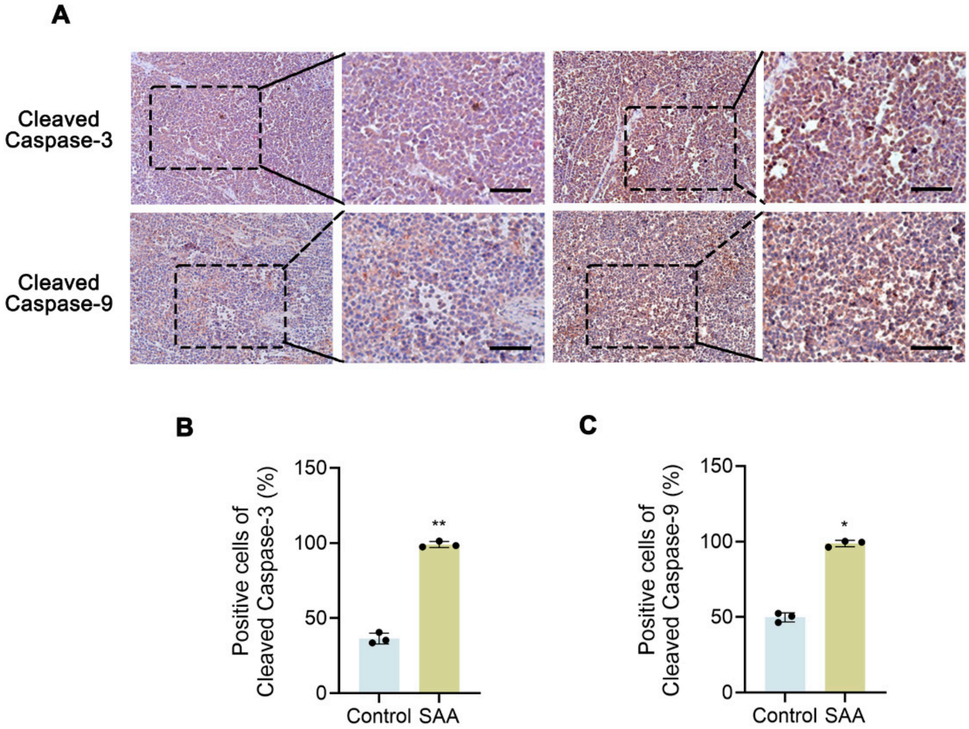

Figure S1 IHC staining of (A,B,C) Cleaved Caspase-3 and Cleaved Caspase-9. Scale bar: 50 μm. ** $p < 0.01$, * $p < 0.05$ vs. control.

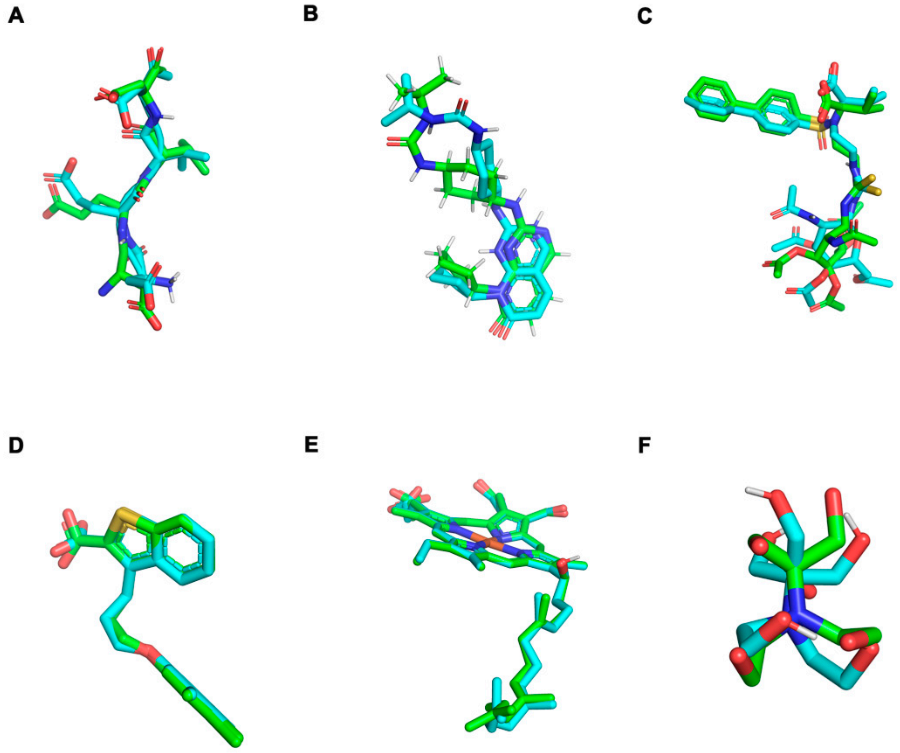

Figure S2 Validation of the docking by redocking of co-crystallized ligands. (A) Redocking validation of Caspase-3 with an RMSD of 0.906 Å. (B) Redocking validation of JUN with an RMSD of 1.166 Å. (C) Redocking validation of MMP9 with an RMSD of 1.48 Å. (D) Redocking validation of BCL-2 with an RMSD of 0.173 Å. (E) Redocking validation of Cytochrome C with an RMSD of 0.056 Å. (F) Redocking validation of SMAC with an RMSD of 1.546 Å.
