# Supplementary material for: Spatial Metabolomics-Based Integrated Analysis Provides Evidence for the Mechanism of Salvianolic Acid A-Induced Mitochondrial Apoptosis in Non-Small Cell Lung Cancer via the ROS-Dependent Caspase Pathway
Source: Biomolecules. 2026 Oct 2;16(10):1434. doi: 10.3390/biom16101434 (PMC13702214; doi:10.3390/biom16101434)

Original Western Blot Images

Figure 7(I)

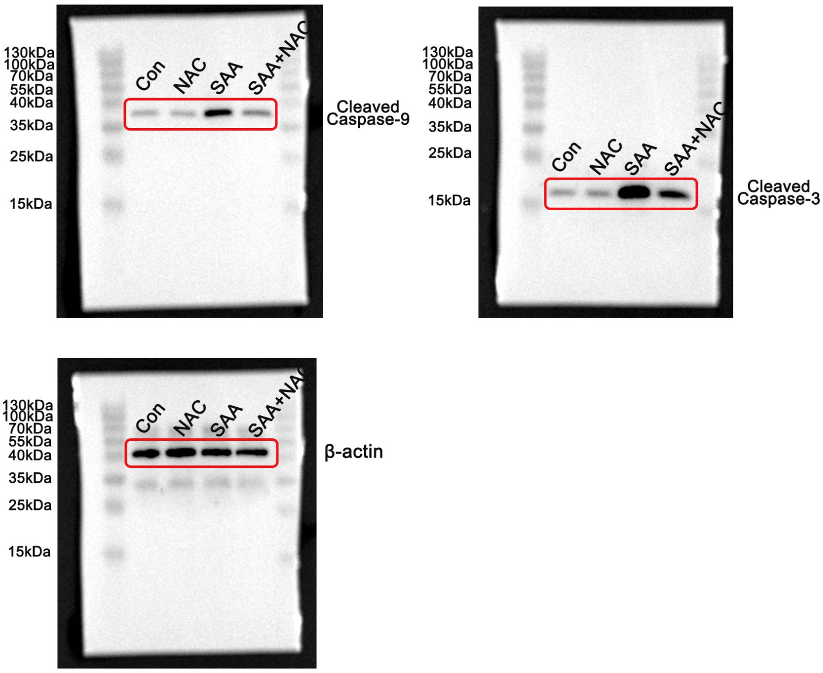

Figure 8(A)

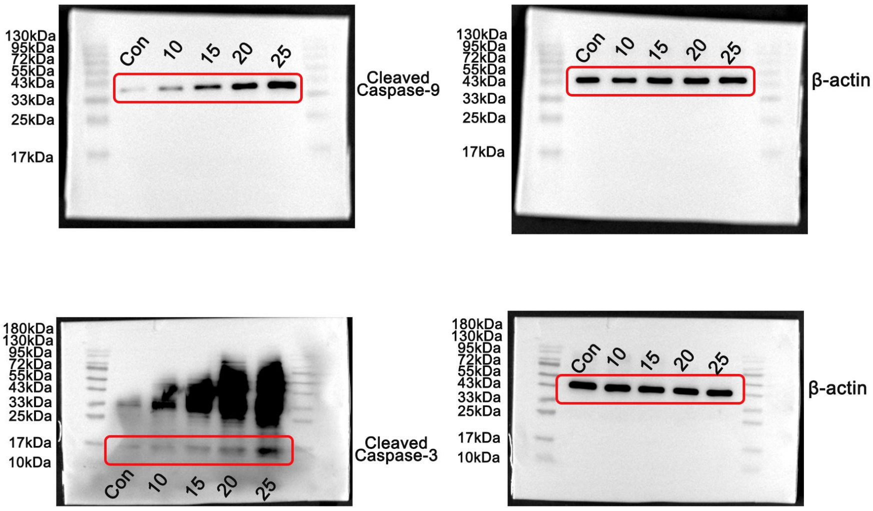

Supplement: Supplementary file 1 [file biomolecules-16-01434-s001.zip › Raw Western Blot Images.pdf]
